# The investigation on shale mechanical characteristics and brittleness evaluation

**DOI:** 10.1038/s41598-023-49934-0

**Published:** 2023-12-22

**Authors:** Wei Lei, Xiangjun Liu, Yi Ding, Jian Xiong, Lixi Liang

**Affiliations:** 1 Petroleum Engineering Technology Research Institute, SINOPEC Southwest Oil & Gas Company, Deyang, 618000 Sichuan People’s Republic of China; 2 State Key Laboratory of Oil and Gas Reservoir Geology and Exploitation, Southwest Petroleum University, Chengdu, 610500 Sichuan People’s Republic of China

**Keywords:** Natural gas, Petrol

## Abstract

Rock mechanical property is significant for shale gas development and exploitation. Shale compressive strength, tensile strength, elastic deformation and so on, are necessary parameters for drilling, completion and fracturing work in shale formation. Among all these shale mechanical parameters, brittleness is a tricky and significant rock property, which has been widely used to hydraulic fracturing design. Currently, although so many works have been conducted to investigate shale brittleness, there is no precise definition of brittleness. In particular, there is no consensus on which method is the most reliable for shale brittleness evaluation. It is vital to figure out how to evaluate shale brittleness in a reliable method. Thus, this paper presents an experimental study on shale mechanical properties, analyzing mechanical features in stress strain curve, relation between mineral content and strength, mechanical parameters at varying confined stress. Based on shale mechanical characteristics and its brittle exhibition, stress strain curve from triaxial compression test is divided into 3 stages, namely, elastic stage, plastic stage and post peak stage. In combined with brittle characteristics in 3 stages of axial and radial stress–strain curves, a new brittleness index has been established for assessing shale brittleness. In order to prove the applicability of new brittleness index, its result is compared with shale failure sample after triaxial test and existing brittleness indexes based on mineral content, elastic deformation, energy, stress and strain, showing a good consistency and proving its practicability. Based on this brittleness index, influence factors of shale brittleness have been discussed. It is shown that elastic module is the most important factor of shale brittleness. Bedding plane makes shale brittleness have strong anisotropy. Brittleness is not only relied on its structure and mineral (like bedding plane, silicate and clay mineral content), but is also highly affected by external stress. Large confined pressure is able to impair shale brittleness. Outcome in this study can offer theoretical guidance for shale exploitation.

## Introduction

As a potential unconventional and clean resource, shale gas has attracted great attention in the whole world. Many countries have conducted their works on shale gas development^1,2^. To efficiently explore shale gas, horizontal drilling and hydraulic fracturing are two vital technologies^3^. Both of these technologies are highly relied on shale mechanical property. For instance, shale compressive strength is associated with wellbore stability in drilling. Especially, shale compressive strength at the wall of borehole is an important factor for drilling design, including drilling fluid density, wellbore trajectory, wellbore configuration and so on^4–6^. For hydraulic fracturing in shale, tensile strength and fracture toughness have huge impact on hydraulic fracture initiation and propagation, finally leading to affect the hydraulic fracturing^7,8^. Therefore, through experimental method and numerical simulation, shale mechanical parameters have been investigated by many researchers to guide drilling and hydraulic fracturing in shale formation^9–11^.

As one of shale mechanical properties, brittleness is a necessary factor for selection of staged fracturing and clustering. High brittleness is the prerequisite for creating effective and complex fracturing network. That is the reason why brittleness is the key parameter in fracturing stimulation. When it comes to brittleness, lots of scholars have studied on its definition, yet there is still no uniform view. All existing brittleness indexes, which have been classified into four types, i.e., methods based on mineral content, elastic deformation, energy, stress–strain, have been demonstrated in Table 1. For brittleness index ($B_{1}$) based on mineral content^12^, $B_{1}$ implies that high brittle mineral content represents strong brittleness. But the limitation is that mineral content doesn’t consider external condition. Rickman et al.^13^ gives brittleness evaluation based on elastic deformation ($B_{2}$). Now, $B_{2}$ is the most widely used methods in oilfield practice. $B_{2}$ uses Young modulus and Poisson ratio to assess shale brittleness. It indicates that shale with high Young modulus and low Poisson ratio has strong brittleness. Whereas, $B_{2}$ is merely based on statistical results. In particular, elastic parameters are unable to exhibit brittle characteristic in plastic stage or post peak stage. Additionally, in fracture mechanics, brittle material is characterized by high resistance of deformation and small resistance of fracturing. Brittleness is the exhibition of shale mechanical property. Thus, based on stress strain curve that contains many rock mechanical information, numerous brittle indexes have been established. Altindag^14^, Bishop et al.^15^, Altindag and Guncy^16^, Fan et al.^17^ use shale stress (peak compressive stress and tensile stress) to establish brittle indexes ($B_{3}$, $B_{4}$, $B_{5}$, $B_{6}$, $B_{7}$, $B_{8}$). Even though those stress parameters are able to reflect brittleness to some extent, they are all acquired from the peak point at loading or post point after failure, which neglects brittle feature before peak point, thus probably making some errors in brittleness evaluation. Similarly, Hajia^18^, Hucka and Das^19^ use strain to build brittle indexes ($B_{9}$, $B_{10}$. To further obtain rock mechanical property from stress strain, brittleness indexes based on energy evolution during rock failure have been given^20,21^. These indexes still can not express the brittleness features at the whole process of stress strain curves. Given different definitions, so far more than 20 methods have been proposed to quantify brittleness^22–25^. Since various methods exist and depend on different theories, it is very difficult to confirm which one is more accurate. Those uncertainties inevitably cause errors in quantifying brittleness in the application.

**Table 1:** Brittleness evaluation methods.

Type	Evaluation methods	Variable declaration	Reference
Method based on mineral content	$B_{1} { = }\frac{{W_{Q} }}{{W_{T} }}$	$W_{Q}$,$W_{T}$ are brittle mineral weight and total mineral weight	Jarvie et al.^12^
Method based on elastic deformation	$B_{2} { = }\frac{{E_{{\text{n}}} + \mu_{n} }}{2}$	$E_{{\text{n}}}$,$\mu_{n}$ are normalized Young modulus and Poisson ratio	Rickman et al.^13^
Method based on stress	$B_{3} { = }\frac{{\sigma_{c} }}{{\sigma_{t} }}$	$\sigma_{c}$,$\sigma_{t}$ are compressive strength and tensile strength	Hucka and Das^19^
	$B_{4} { = }\frac{{\sigma_{{\text{c}}} - \sigma_{t} }}{{\sigma_{{\text{c}}} + \sigma_{t} }}$		Hucka and Das^19^
	$B_{5} { = (}\sigma_{t} \sigma_{c} )^{0.5} /2$		Altindag^14^
	$B_{6} { = }\frac{{\sigma_{P} - \sigma_{r} }}{{\sigma_{P} }}$	$\sigma_{P}$,$\sigma_{r}$ are peak and residual strength	Bishop^15^
	$B_{7} { = }\sqrt {\sigma_{T} }$	$\sigma_{T}$ is maximum tensile strength	Altindag and Guncy^16^
	$B_{8} { = }\frac{{V_{c} }}{{V_{I} }}$	V_c_ and V_I_ are parameters from Marinell hardness of nano-indentation method	Fan et al.^17^
Method based on strain	$B_{9} { = }\frac{{\varepsilon_{P} - \varepsilon_{r} }}{{\varepsilon_{P} }}$	$\varepsilon_{P}$,$\varepsilon_{r}$ are peak and residual strain	Hajia and Peter^18^
	$B_{10} { = }\frac{{\varepsilon_{el} }}{{\varepsilon_{tot} }}$	$\varepsilon_{el}$, $\varepsilon_{tot}$ are elastic and total strain at failure, respectively	Hucka and Das^19^
Method based on energy	$B_{11} = \frac{{U^{f} }}{{U_{c}^{e} }}$	$U^{f}$, $U_{c}^{e}$ are total failure energy and consumed elastic strain energy during the failure process, respectively	Tarasovn and Randolph^20^
	$B_{12} = \frac{{U^{a} }}{{U_{c}^{e} }}$	$U^{a}$ is the additional input energy	Tarasovn and Potvin^21^
	$B_{13} = \frac{{U^{a} }}{{U_{c}^{e} + U_{p}^{d} }}$	$U_{p}^{d}$ is peak dissipated energy	Ai et al.^26^
Others	$B_{14} { = }\frac{H}{{K_{IC} }}$	$H$ is hardness and $K_{IC}$ is fracturing toughness	Lawn and Marshall^22^
	$B_{15} { = }\frac{{\sigma_{1} - \sigma_{3} }}{{2C\cos \varphi + (\sigma_{1} + \sigma_{3} )\sin \varphi }}$	$C$, $\varphi$ are cohesion and internal friction angle. $\sigma_{1}$, $\sigma_{3}$ are maximum and minimum principal stress	Papanastasiou et al.^23^
	$B_{16} { = }0.198\sigma_{c} - 2.174\sigma_{t} + 0.913\rho - 3.807$	$\rho$ is rock density	Yagiz^24^
	$B_{17} {\text{ = e}}^{{\frac{E}{10M} \times \frac{{\sigma_{p} - \sigma_{r} }}{{\sigma_{p} }} \times \sin \varphi }}$	M is post-peak modulus, GPA	Zhou et al.^25^

It is clear from the literature review that numerous researches utilize various kinds of parameters to assess brittleness, but most of their studies only consider the brittle property from one aspect, lack of comprehensive analysis, which will impair its precision. Although there are lots of studies about brittleness evaluations on the basis of stress strain curve, these methods are depended on axial stress strain curve. The brittle performance not only shows up at axial deformation, but also exists in radial deformation. Therefore, it is significant to have further research on shale brittleness evaluation. In this study, shale mechanical characteristics has been acquired using triaxial test. In addition, considering brittle performance in elastic stage, plastic stage, post-peak stage at axial and radial stress–strain curve, a new brittle index has been built. According to this new brittle index, influence factors of shale brittleness have been fully discussed. The outcome of this paper can improve the understanding of shale brittleness and is meaningful for shale gas development.

## Shale sample

**Figure 1:**
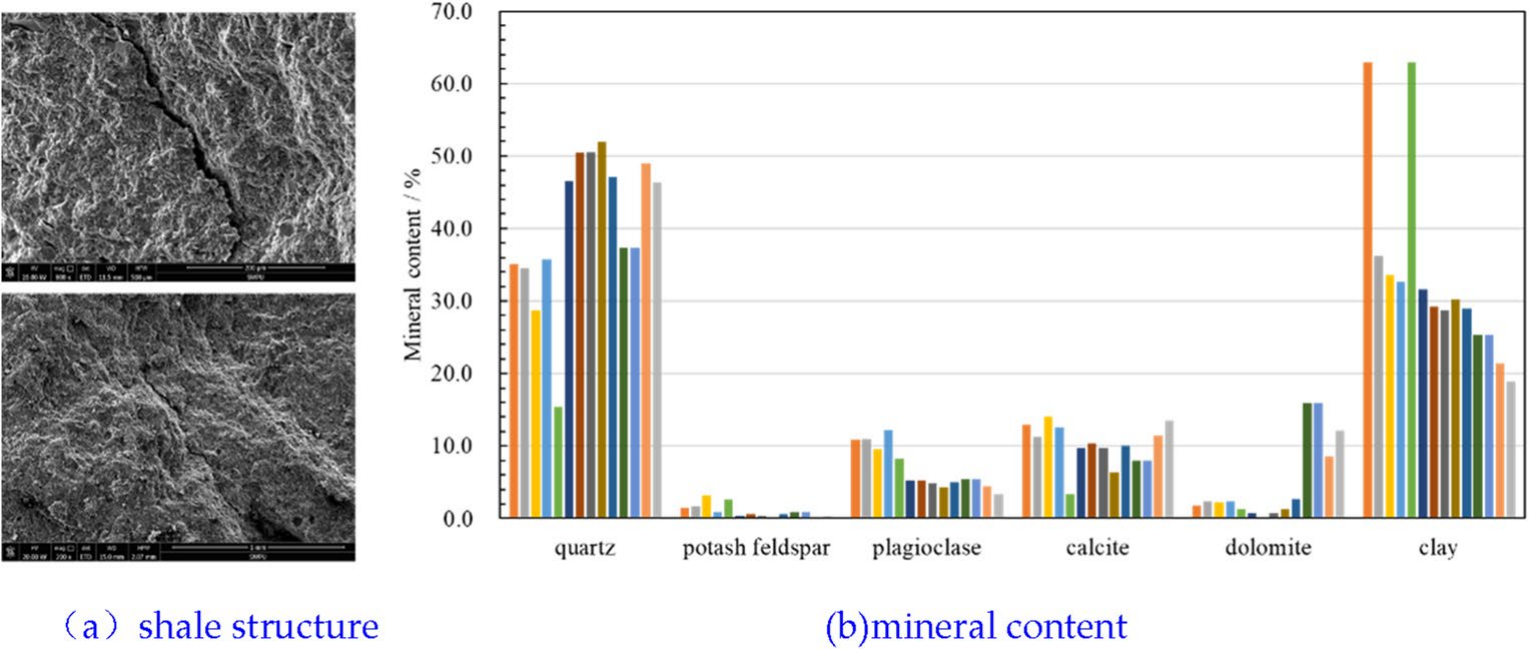
Shale micro-structure and mineral content.

Shale sample has been acquired from Longmaxi formation, Sichuan basin (depth is located at 4390–4420 m). SEM test illustrates shale of Longmaxi formation is rich with microfractures and micropores, shown as Fig. 1a. According to XRD test results (Fig. 1b), clay and quartz are dominated in shale, with 34.6% and 40.5% average content. Also, brittle minerals, such as quartz, potash feldspar and plagioclase, are over 50% of mineral content, showing strong brittleness of shale.

In addition, core samples have been used for rock physical test, including density, acoustic property, porosity, etc., shown as Table 2. Density and porosity of shale are 2.53–2.66 g/cm^3^ and 1.21–7.51% respectively. Interval transit time of P wave and S wave are 193.56–207.58 us/ft and 332.43–354.18 us/ft. Based on rock physical property of shale, samples are selected and prepared for following triaxial test.

**Table 2:** Rock physical property of shale sample.

No	Depth/m	Length/mm	Diameter/mm	Density/g cm^3^	Porosity/%	P wave/μs ft	S wave/μs ft
1	4388.68	50.63	24.96	2.66	3.8	193.56	333.40
2	4392.69	50.54	25.05	2.65	1.8	199.85	332.43
3	4396.45	50.39	25.06	2.64	2.17	203.20	338.14
4	4396.71	50.32	25.04	2.64	1.38	197.77	335.06
5	4397.60	50.30	25.08	2.65	3.82	197.61	334.00
6	4403.14	50.58	25.18	2.56	1.22	203.64	336.10
7	4407.25	50.57	25.24	2.56	6.39	201.29	335.35
8	4408.91	50.15	25.20	2.55	2.30	201.79	335.00
9	4409.88	50.06	25.14	2.53	4.69	204.97	337.22
10	4412.35	50.20	25.10	2.54	2.67	207.58	334.68
11	4412.41	50.31	25.09	2.55	2.76	201.56	343.49
12	4414.67	50.20	25.15	2.53	7.51	202.78	350.57
13	4415.41	50.37	25.22	2.53	2.55	205.68	354.18
14	4416.63	50.21	25.16	2.53	1.91	200.35	345.72

## Shale mechanical characteristics

### Triaxial test

**Figure 2:**
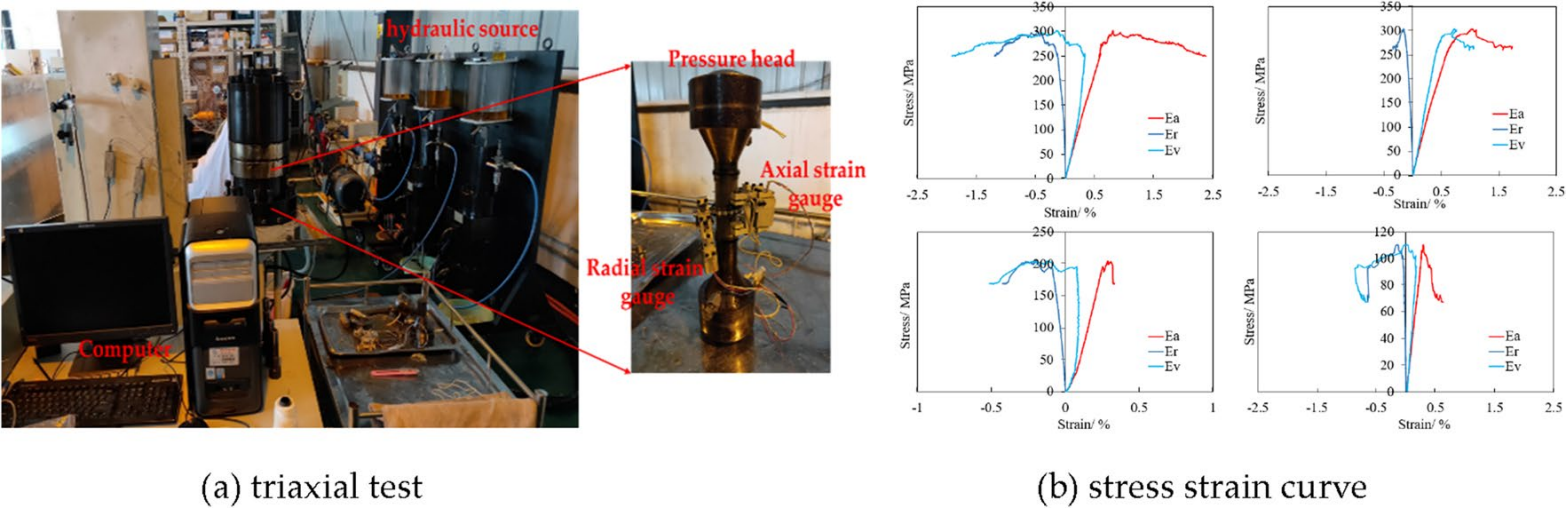
Triaxial test and its stress strain curve of shale.

In this paper, shale mechanical characteristics is exhibited by conducting triaxial test, shown as Fig. 2a. Based on that, stress strain curves with certain confined stress have been acquired, shown as Fig. 2b. Stress strain curve is able to give mechanical parameters, such as compressive strength (peak stress), elastic module, Poisson ratio, which are all indicators of shale rock mechanical characteristics.

### Relation between mineral content and mechanical parameters

**Figure 3:**
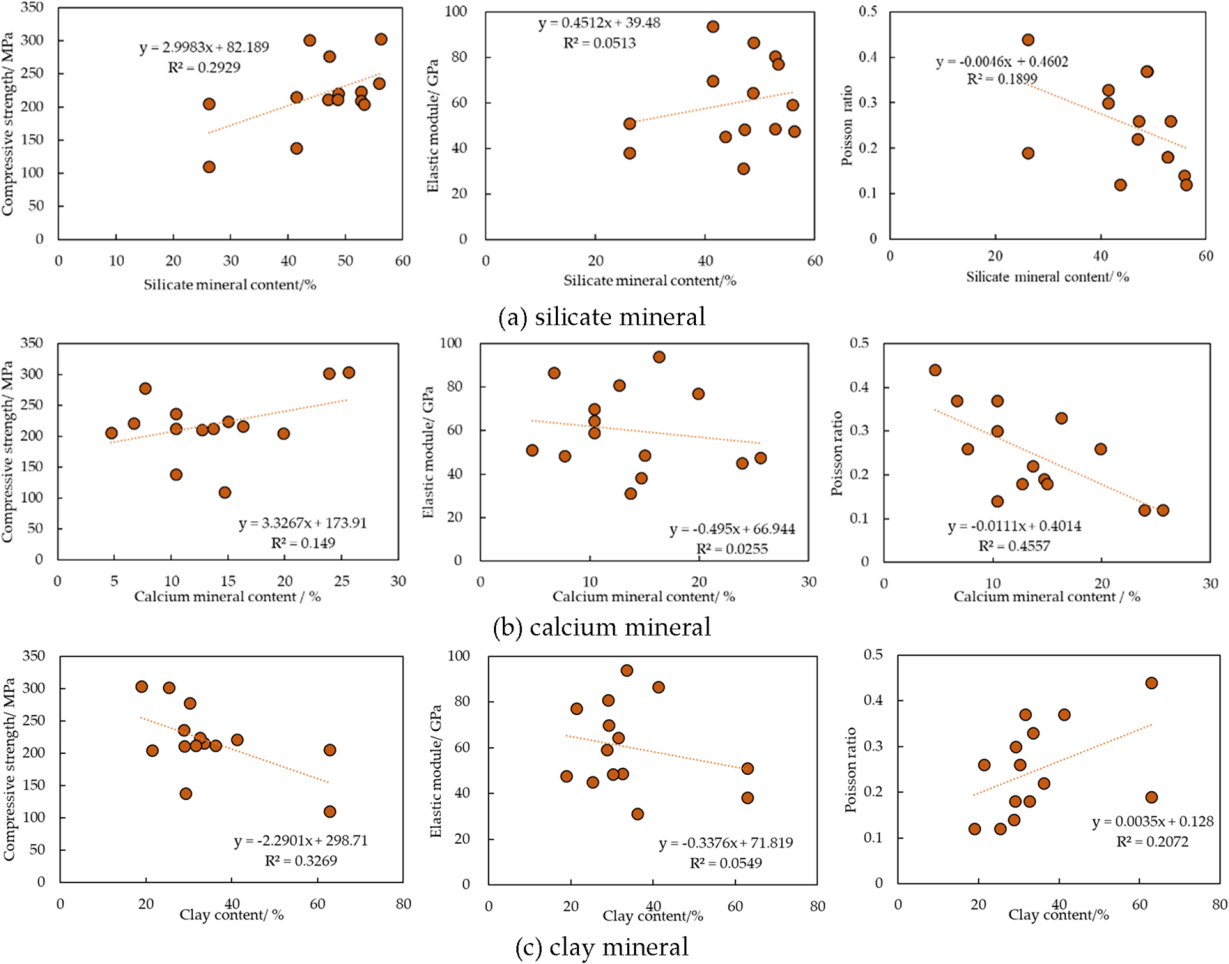
Relation between mineral content and mechanical parameters.

In order to obtain the association between mineral content and mechanical parameters, triaxial test and XRD have been combined. After triaxial test, shale fragments were collected for mineral content analysis. Results are shown as Fig. 3. Minerals are classified to 3 types, which are silicate (quartz, potash feldspar, plagioclase), calcium (calcite, dolomite) and clay mineral. It can be found that compressive strength has decline with increasing silicate and calcium mineral since they (quartz, potash feldspar) are comparatively strong strength mineral. On the other hand, clay as relatively lower strength mineral, is adverse to shale strength. Since silicate mineral is brittle mineral, high content of silicate mineral leads to large elastic module and small Poisson ratio, which are typical brittle features according to Rickman^13^. In contrast, clay normally has plastic property. Its increasing content causes small elastic module and large Poisson ratio.

### Influence of confined stress on shale mechanical characteristics

**Figure 4:**
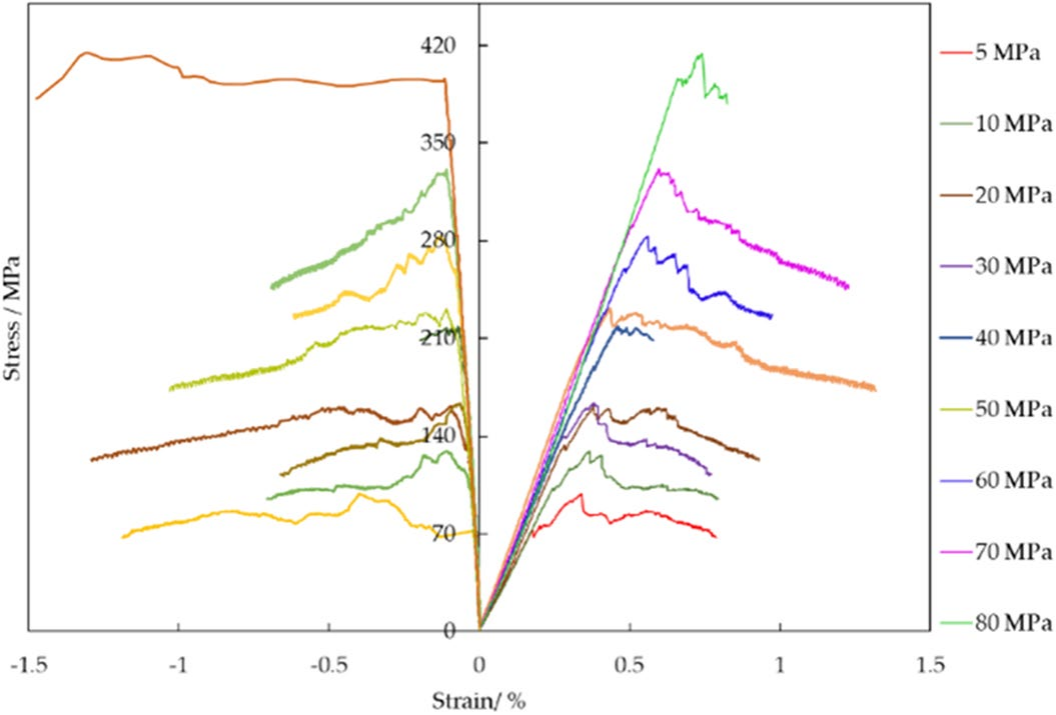
Stress strain curves with different confined pressure.

**Figure 5:**
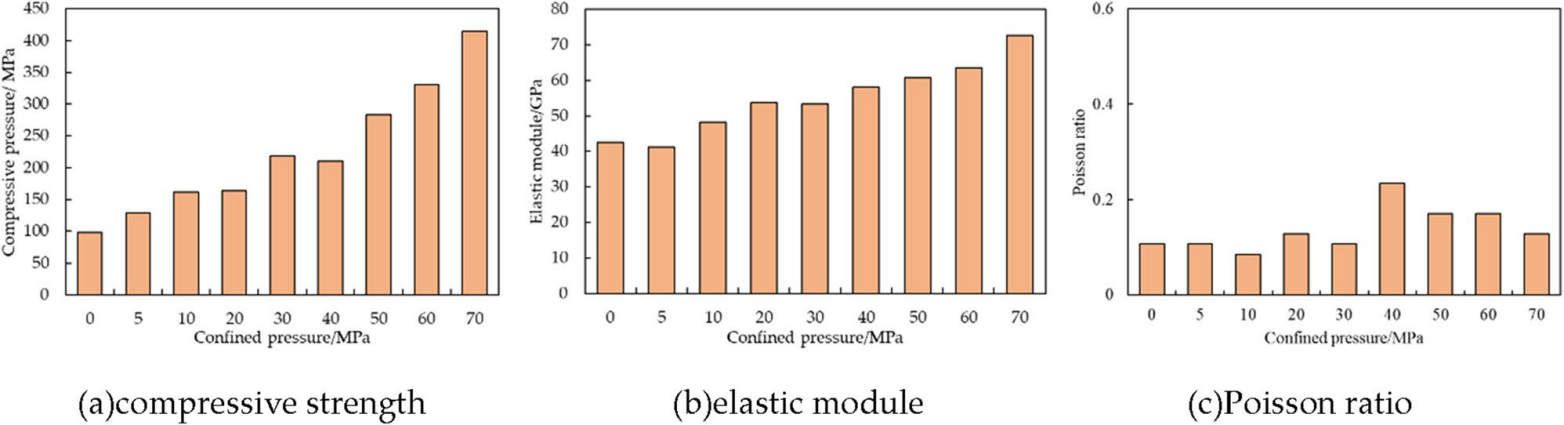
Shale rock mechanical parameters with different confined pressure.

To further analyze shale mechanical characteristics in different stress conditions, triaxial tests with various confined pressures have been conducted, and its result is shown in Fig. 4. Furthermore, based on stress strain curves with different confined pressures, compressive strength, elastic module and Poisson ratio have been illustrated in Fig. 5. It is shown that confined pressure can increase compressive strength because it adds up lateral braced force. With rising confined pressure, elastic module has growing trend and no clear change of Poisson ration is noticed.

### Influence of bedding plane on shale mechanical characteristics

**Figure 6:**
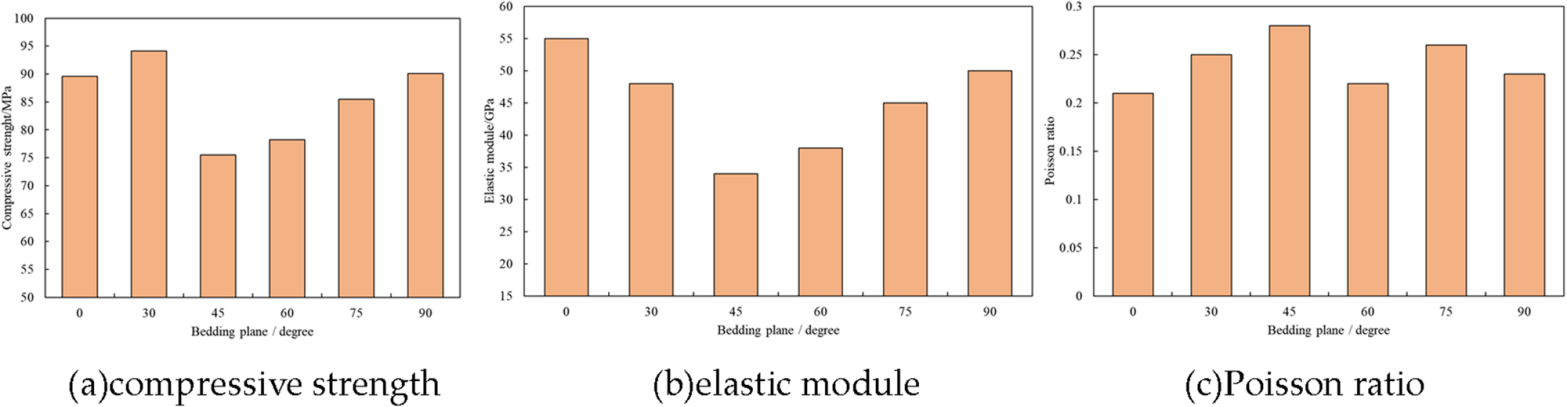
Shale rock mechanical parameters with different bedding plane angles.

Shale is typical laminated material with parallel bedding plane, which normally has weak strength^10^. In order to analyze the influence of weak plane on shale mechanical property, shale samples with variable bedding plane angles have been applied to triaxial test. Results are shown as Fig. 6. With increasing bedding plane angle, shale strength and elastic module firstly decline and reach lowest point at approximately 55°. Then, shale strength and elastic module rise back to original value. In contrast, varying bedding plane angles have little impact on Poisson ratio.

## Shale brittleness

### New brittleness index based on stress strain curve

It has been known that brittleness is largely relied on stress strain curve. Correspondingly, many brittleness indexes have been established based on stress strain curve^27^. However, few evaluation methods can cover the whole process of stress strain curve, mainly focus on elastic stage or post-peak stage. Brittleness is exhibited in the overall process of rock failure, thus evaluation based on whole process of stress strain curve is necessary. In addition, most of brittle evaluations are built on axial stress–strain curve, ignoring the radial stress–strain curve that is an important indicator of rock brittleness.

**Figure 7:**
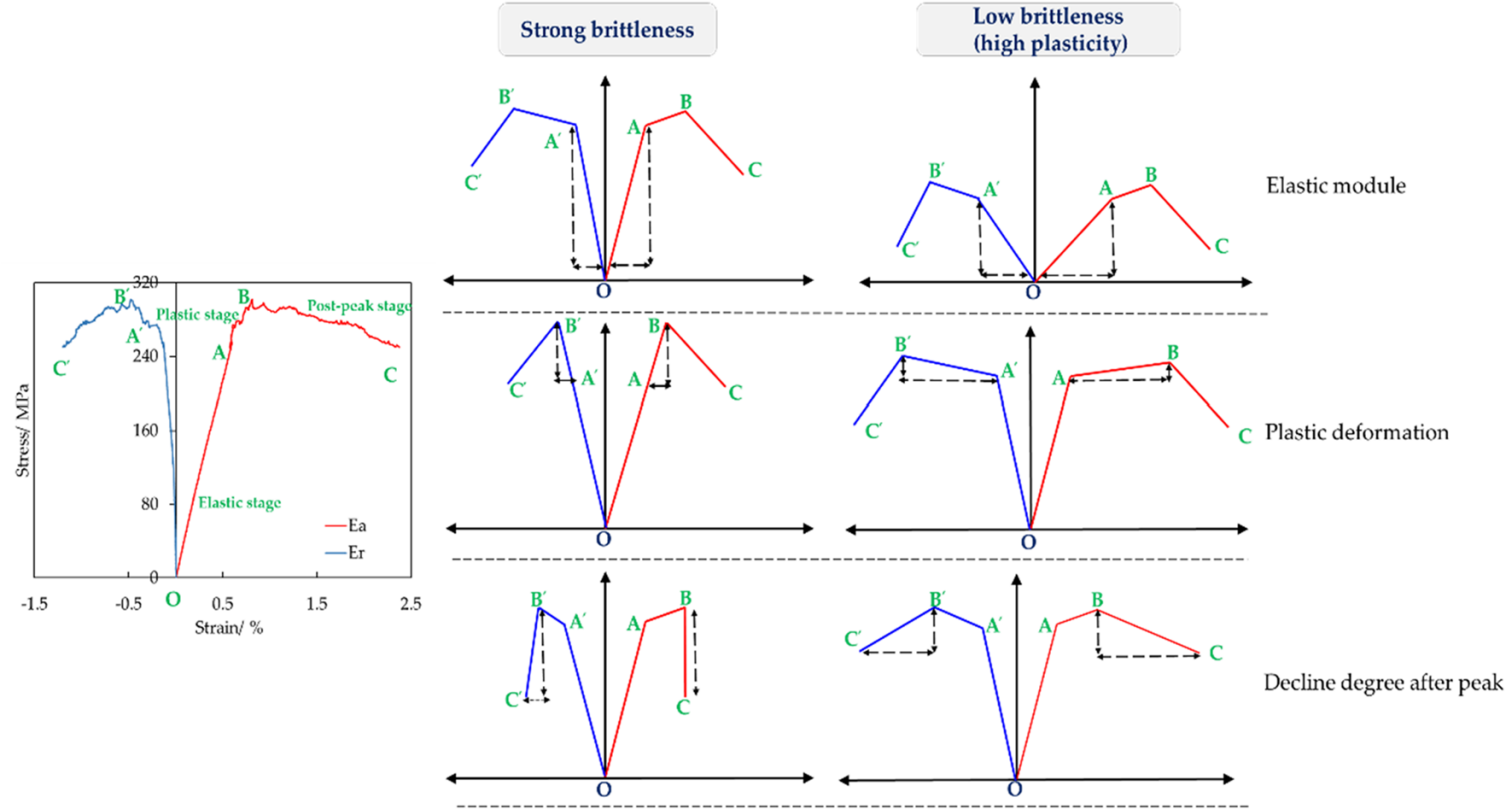
Schematic of shale brittleness index.

According to rock mechanical theory and existing brittleness evaluation, typical brittle characteristics in stress strain curves are listed as^28–30^: (1) few elastic deformation (large elastic module),(2) plastic stage is short; (3) stress has dramatic dropping after peak point; (4) small ratio of radial to axial deformation. All these features are in stress strain curve shown as Fig. 7. Based on Fig. 7, for axial and radial stress–strain curve, they all can be divided into 3 stages, i.e., elastic stage, plastic stage and post-peak stage. Based on above brittle characteristics, angles of inclination of stress–strain curves in each stage have bee applied to build brittle index, shown as:

1 $$\left\{ \begin{gathered} BI_{a} = \arctan \frac{{\sigma_{A} }}{{\varepsilon_{A} }} + \arctan \frac{{\sigma_{B} - \sigma_{A} }}{{\varepsilon_{B} - \varepsilon_{A} }} + 180^\circ - \arctan \frac{{\varepsilon_{C} - \varepsilon_{B} }}{{\sigma_{B} - \sigma_{C} }} \hfill \\ BI_{r} = \arctan \frac{{\sigma_{{A^{\prime} }} }}{{ - \varepsilon_{{A^{\prime} }} }} + \arctan \frac{{\sigma_{{B^{\prime} }} - \sigma_{{A^{\prime} }} }}{{\varepsilon_{{A^{\prime} }} - \varepsilon_{{B^{\prime} }} }} + 180^\circ - \arctan \frac{{\varepsilon_{{C^{\prime} }} - \varepsilon_{{B^{\prime} }} }}{{\sigma_{{C^{\prime} }} - \sigma_{{B^{\prime} }} }} \hfill \\ \end{gathered} \right.$$

where $BI_{a}$, $BI_{r}$ are brittle indexes based on axial stress–strain curve and radial stress–strain curve. $\sigma_{A}$ and $\varepsilon_{A}$ are stress and strain at point A. $\sigma_{B}$ and $\varepsilon_{B}$ are stress and strain at point B. $\sigma_{C}$ and $\varepsilon_{C}$ are stress and strain at point C. $\sigma_{{A^{\prime} }}$ and $\varepsilon_{{A^{\prime} }}$ are stress and strain at point A^’^. $\sigma_{{B^{\prime} }}$ and $\varepsilon_{{B^{\prime} }}$ are stress and strain at point B^’^. $\sigma_{{C^{\prime} }}$ and $\varepsilon_{{C^{\prime} }}$ are stress and strain at point C^’^.

In addition, small ratio of radial to axial deformation represents high brittleness, thus giving a brittle index:

2 $$BI_{ar} = \left| {\frac{{\sigma_{A} /\varepsilon_{A} }}{{\sigma_{{A^{\prime} }} /\varepsilon_{{A^{\prime} }} }}} \right|$$

In combination with Eq. (1) and Eq. (2), a new brittleness index including all brittle characteristics in stress strain curves has been established, shown as:

3 $$BI = \frac{1}{3}\left( {\frac{{BI_{a} - BI_{a\min } }}{{BI_{a\max } - BI_{a\min } }} + \frac{{BI_{r\max } - BI_{r} }}{{BI_{r\max } - BI_{r\min } }} + \frac{{BI_{ar} - BI_{ar\min } }}{{BI_{ar\max } - BI_{ar\min } }}} \right)$$

where $BI$ is final brittleness index. $BI_{a\max }$,$BI_{a\min }$ are maximum and minimum $BI_{a}$. $BI_{r\max }$,$BI_{r\min }$ is maximum and minimum $BI_{r}$. $BI_{ar\max }$,$BI_{ar\min }$ are maximum and minimum $BI_{ar}$.

### Verification of new brittleness index

**Figure 8:**
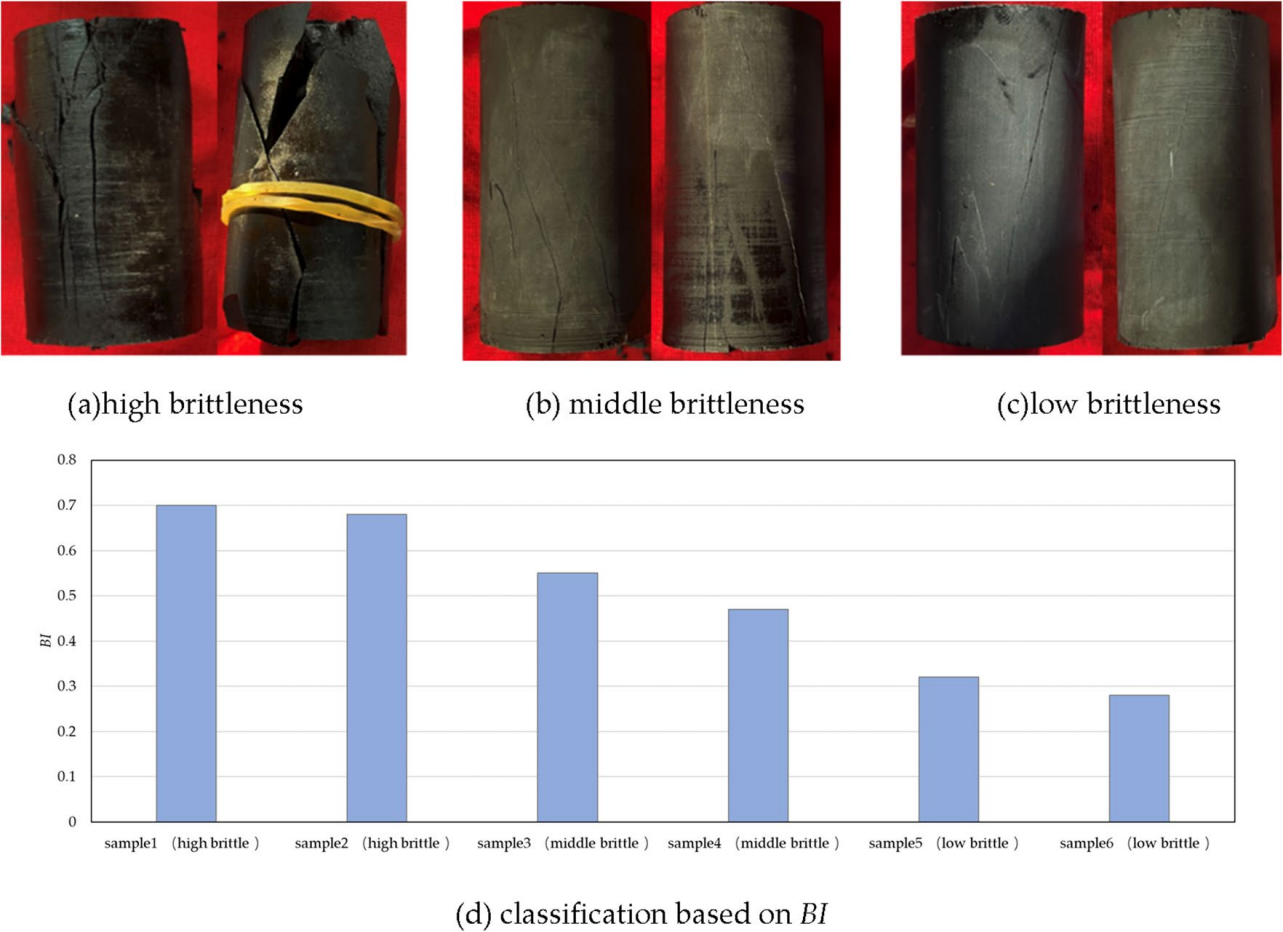
Brittleness classification of shale in Longmaxi reservoir.

**Figure 9:**
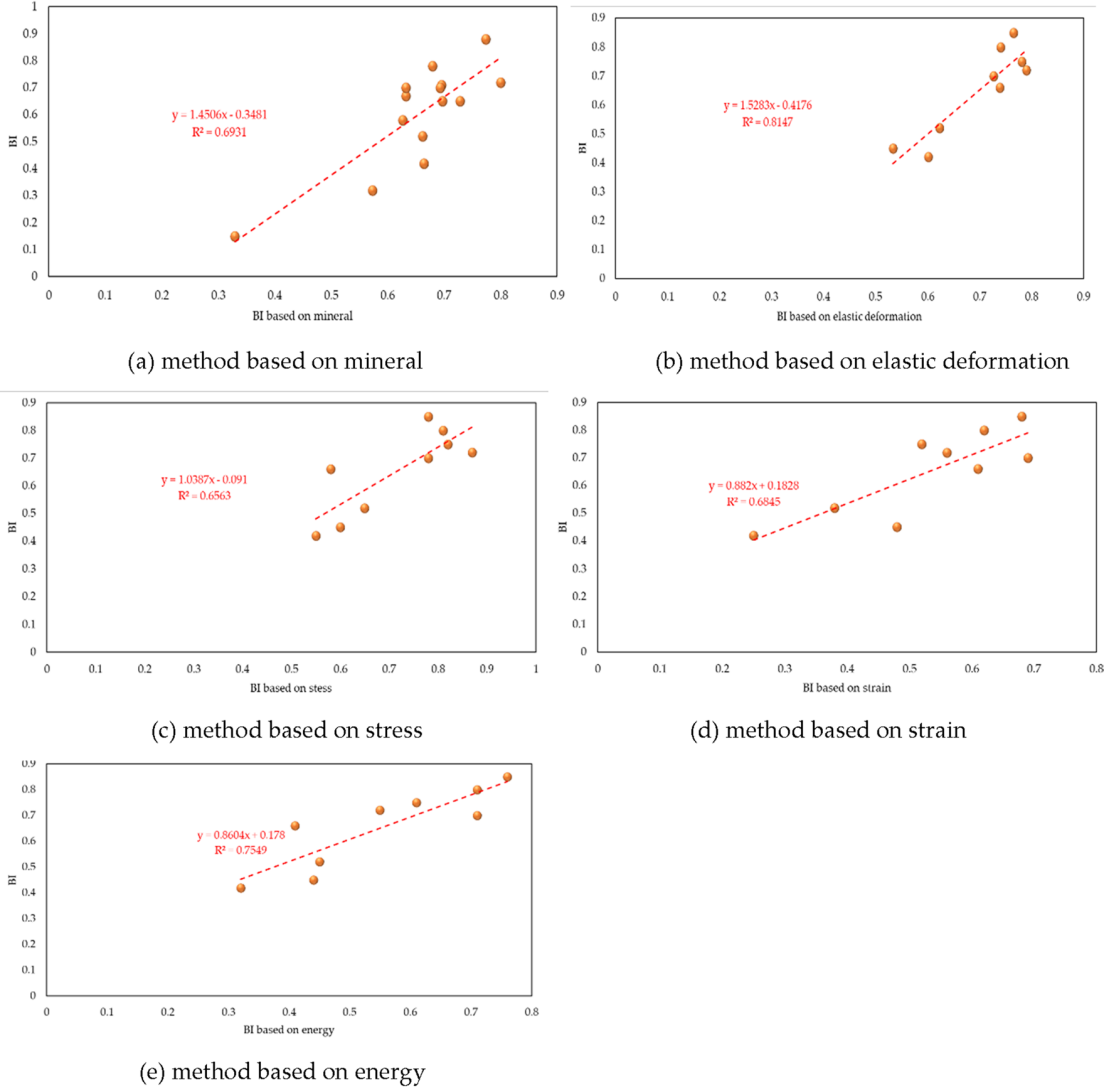
Relation of *BI* and existing brittleness method.

In order to verify this brittleness index, two comparisons have been done. Firstly, rock with high brittleness indicates strong damaging degree after failure, which is also the reason why brittleness is an important factor of hydraulic fracturing^31–33^. Classifications based on failure degree of shale sample and *BI* have been illustrated respectively in Fig. 8. Secondly, existing brittleness methods, which are established on mineral content (*BI*_*1*_), elastic deformation(*BI*_*2*_), stress(*BI*_*6*_), strain (*BI*_*8*_) and energy (*BI*_*12*_) respectively, have been selected. The relation between *BI* and existing brittleness methods have been given, shown as Fig. 9.

It is shown that brittleness classification based on *BI* accords with failure degree of shale sample (Fig. 8d). Samples with multiple cracks and strong degree of fragmentation after triaxial test show high value of *BI*. Besides, samples with single crack and low degree of fragmentation have small value of *BI*. This consistency proves *BI* has a good ability of brittleness prediction. Additionally, *BI* is consistence with five types of existing brittleness (Fig. 9), further proving the accuracy of *BI*.

### Influence factors of shale brittleness

In the oilfield, shale brittleness is evaluated on the logging data, which can not directly give shale stress strain curve. Thus, it is necessary to establish shale brittleness evaluation based on parameters from logging, such as mineral distribution, density, interval transit time (DT), porosity, elastic module, Poisson ratio, confined pressure and bedding plane occurrence^34^. Therefore, in this part, relation between these influence factors and shale brittleness has been discussed.

**Figure 10:**
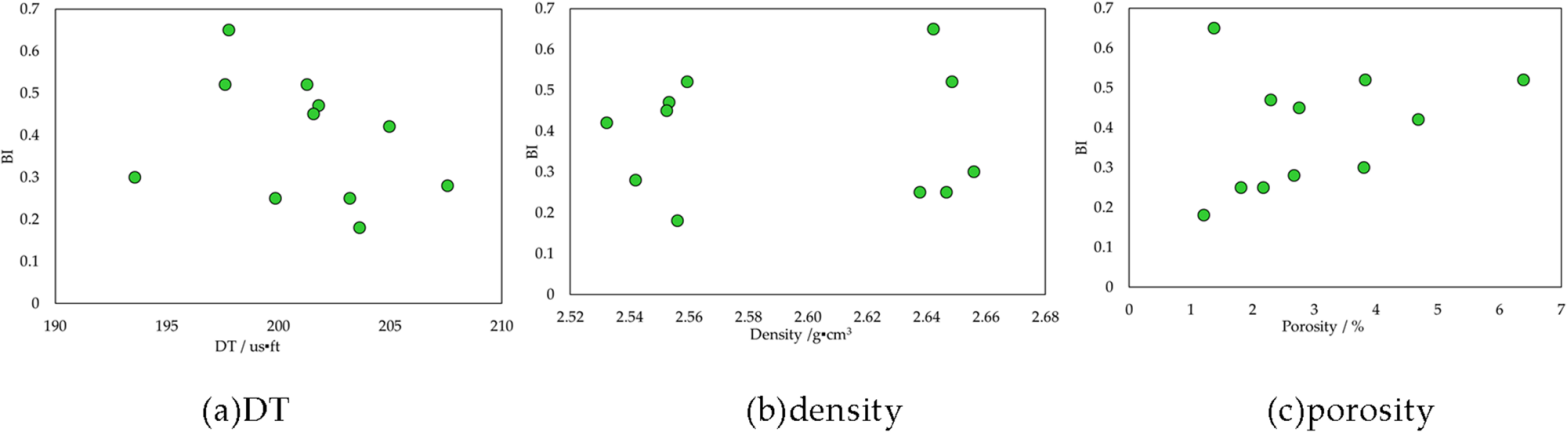
Relation of *BI* and shale physical properties.

First of all, the fundamental logging information are rock physical, such as acoustic interval transit time (DT), density and porosity. Their correlations with shale brittleness are shown as Fig. 10. Shale brittleness has no association with these rock physical parameters, meaning that it is unable to evaluate shale brittleness by using these basic logging data.

**Figure 11:**
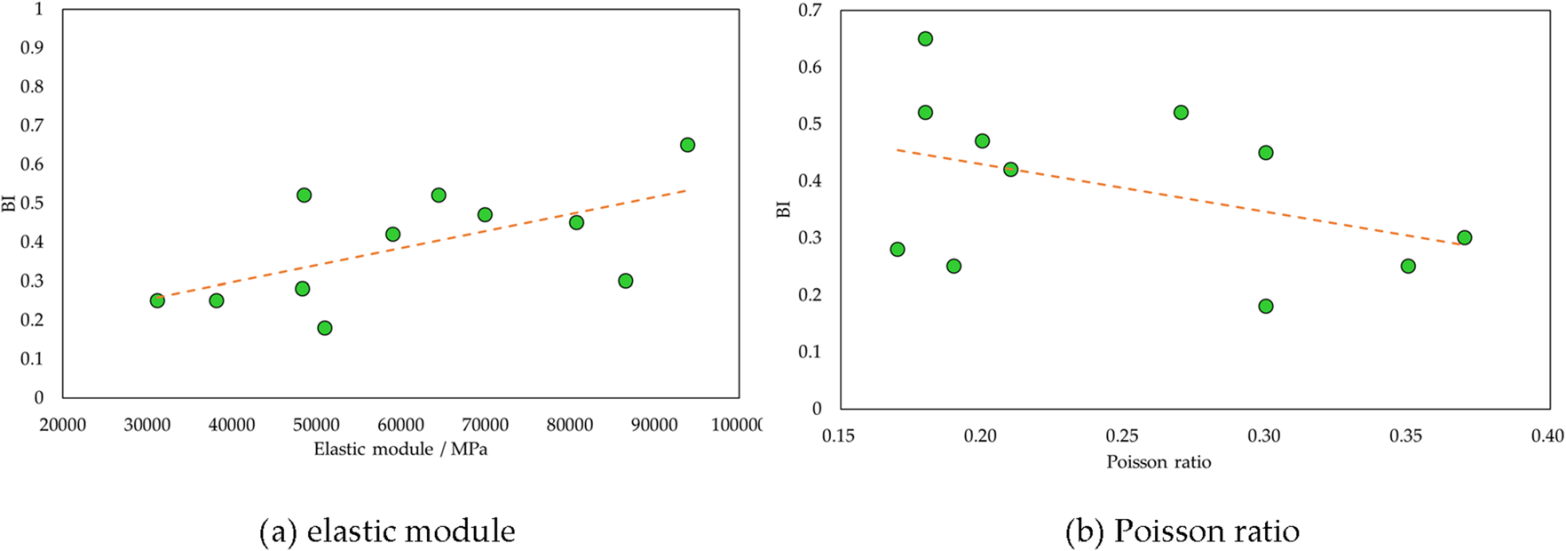
Relation of *BI* and elastic module, Poisson ratio.

In addition, based on sonic wave theory, elastic parameters (elastic module and Poisson ratio) can be acquired using DT and density. Correspondingly, their relations with brittleness have been illustrated in Fig. 11. It is found that with increasing brittleness, elastic module grows and Poisson ratio decreases. This pattern further proves the practicability of Rickman equation and also indicates that elastic parameters are robust factors of evaluating shale brittleness.

**Figure 12:**
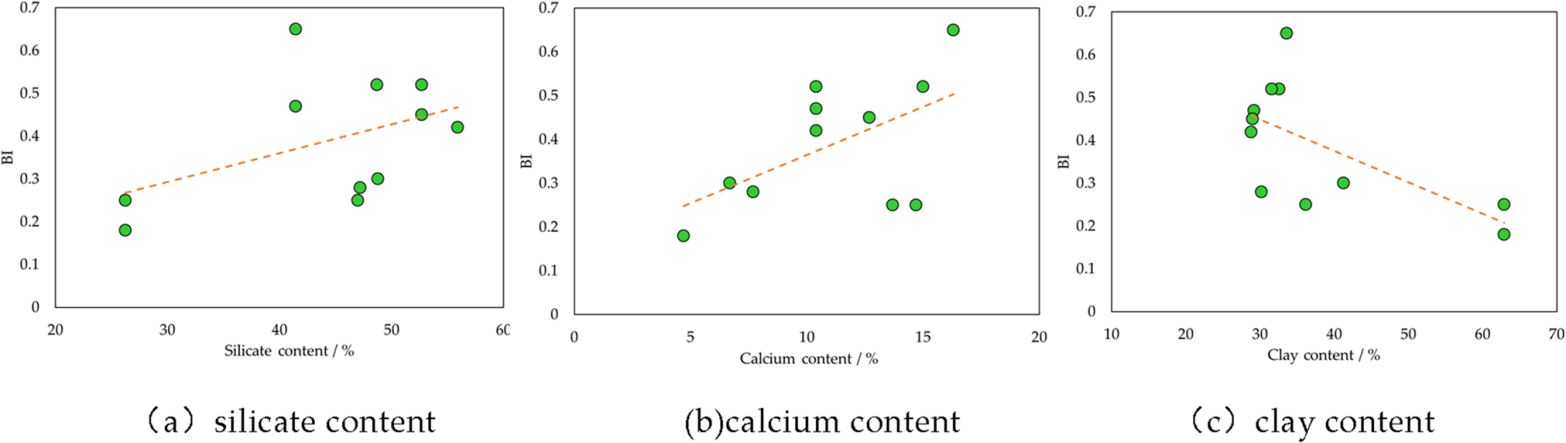
Relation of *BI* and mineral content.

Logging is able to offer profile of mineral content. The relation between mineral content and brittleness is shown in Fig. 12. It demonstrates that silicate and calcium, as typical brittle mineral, are beneficial for shale brittleness. On the other hand, with increasing clay that has small brittleness, shale brittleness shows decline. Results indicate that mineral content is an important factor of shale brittleness.

**Figure 13:**
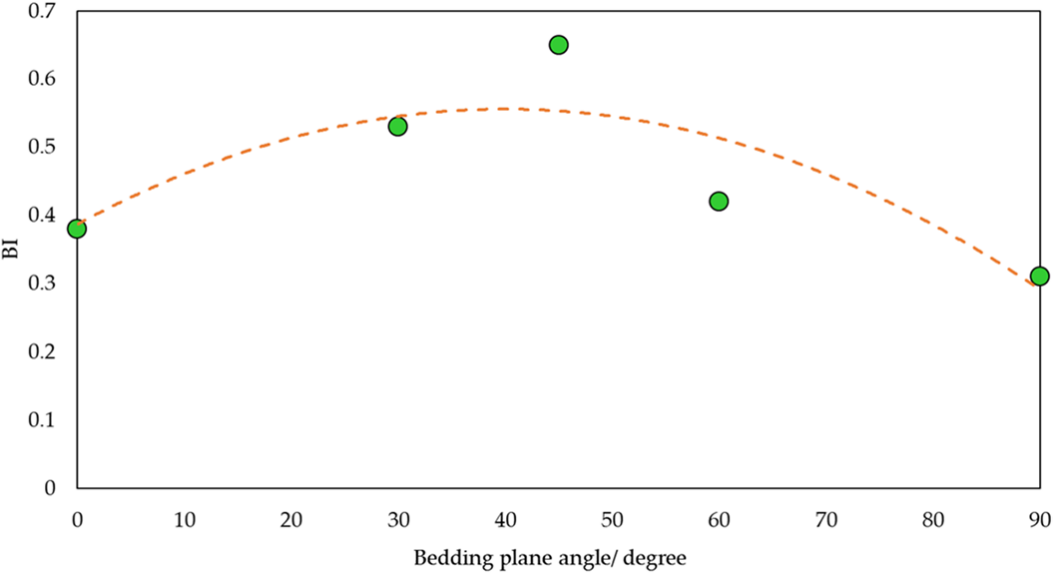
Relation of *BI* and bedding plane.

**Figure 14:**
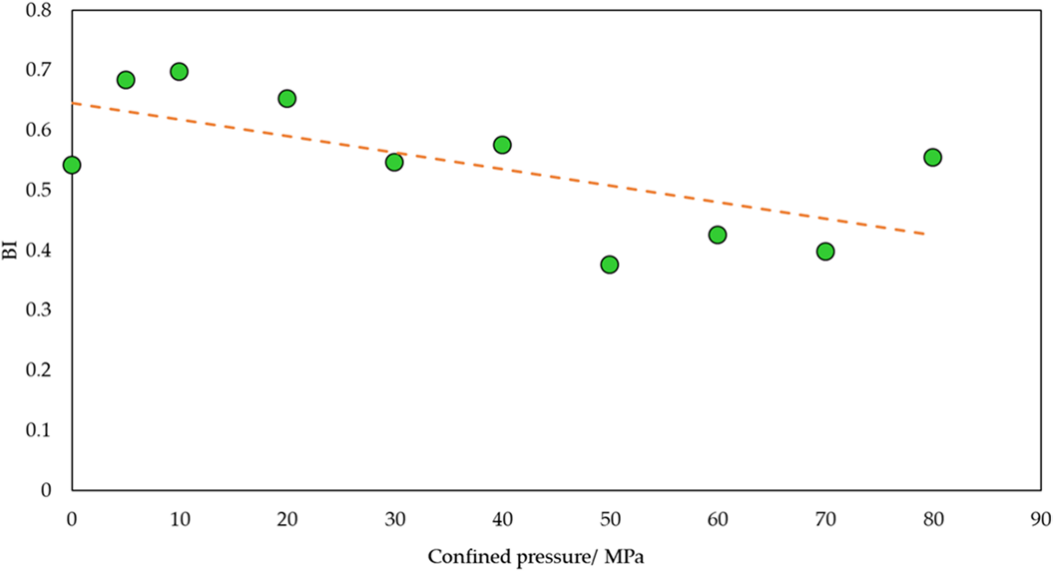
Relation of *BI* and confined pressure.

Bedding plane is a significant structure that can affect rock mechanics. The association between bedding plane occurrence and brittleness is shown in Fig. 13. It is shown that brittleness firstly increases and then drops with rising bedding plane angle. It can be explained by the failure model of shale. Bedding plane is the typical weak strength plane. In this condition, when axial stress acts on shale, it is likely to have failure along bedding plane. With shale failure along bedding plane, its strength is small. Axial stress can cause stronger and fast structural damage, thus expressing high brittleness. Furthermore, shale brittleness is not depended on its physical, mechanical property, mineral content and structure, it is also affected by external stress. Therefore, relation between confined pressure and brittleness has been illustrated in Fig. 14. Since confined pressure has restriction on strain and boost impact on strength, meaning that it is not easy to create crack and make sample failure during mechanical test. Thus, this small failure degree express low brittleness.

According to above analysis, elastics module, Poisson ratio, silicate content, calcium content, clay content, bedding plane angle and confined pressure are all important influence factors of shale brittleness. In order to quantify the importance degree of all influence factors, normalization processing has been applied to all influence factors, shown as:

4 $$\left\{ \begin{gathered} Y(i) = \frac{{x(i) - X_{\min } (i)}}{{X_{\max } (i) - X_{\min } (i)}}\;\;\;\;positive\;correlation \hfill \\ Y(i) = \frac{{X_{\max } (i) - x(i)}}{{X_{\max } (i) - X_{\min } (i)}}\;\;\;\;negative\;correlation \hfill \\ \end{gathered} \right.\;\;i = 1,2,3....n$$

where $x(i)$ is value of influence factor.*i*. $X_{\max } (i)$, $X_{\min } (i)$ are maximum and minimum $x(i)$ $Y(i)$ is normalization of $x(i)$.

After normalization, changing coefficient of influence factor *i* ($Z(i)$) has been obtained :

5 $$Z(i) = \frac{{BI_{\max } (i) - BI_{\min } (i)}}{{Y(i)_{\max } - Y(i)_{\min } }}$$

where $BI_{\max } (i)$, $BI_{\min } (i)$ are maximum and minimum brittleness index with changing $Y(i)$. $Y(i)_{\max }$, $Y(i)_{\min }$ are value of $Y(i)$ when brittleness indexes are maximum and minimum respectively.

**Figure 15:**
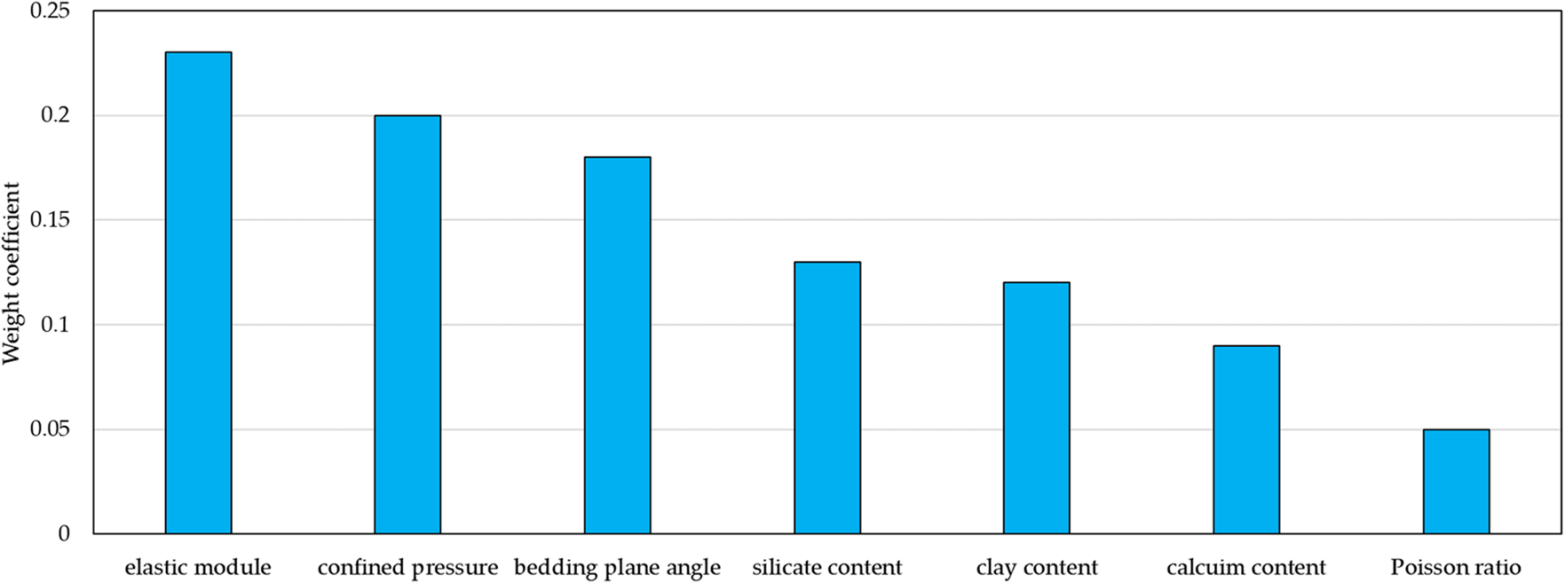
Weight coefficients of all influence factors of shale brittleness.

Obviously, larger $Z(i)$ indicates that influence factor *i* ($x(i)$) has stronger impact on brittleness index. Hence, based on $Z(i)$, weight coefficients of all influence factors are computed using Eq. (6), illustrated as Fig. 15.

6 $$\delta (i) = \frac{Z(i)}{{\sum\limits_{i = 1}^{n} {Z(i)} }}\;\;\;\;\;i = 1,2,3.....n$$

It is shown that elastic module has highest weight coefficients, but the influence of Poisson ratio is smallest. Mineral content plays a big role in shale brittleness. In particular, silicate and clay mineral content are directly linked to brittleness. Weight coefficient of confined pressure is large, indicating external condition is vital for shale brittleness evaluation. Also, it indicates shale brittleness has decline when shale gas exploitation goes into deep shale formation with high stress. Due to rich bedding plane, shale has strong anisotropy in brittleness. Since weight coefficient of bedding plane angle is so high, it is necessary to consider bedding plane occurrence in shale brittleness evaluation.

## Application

**Figure 16:**
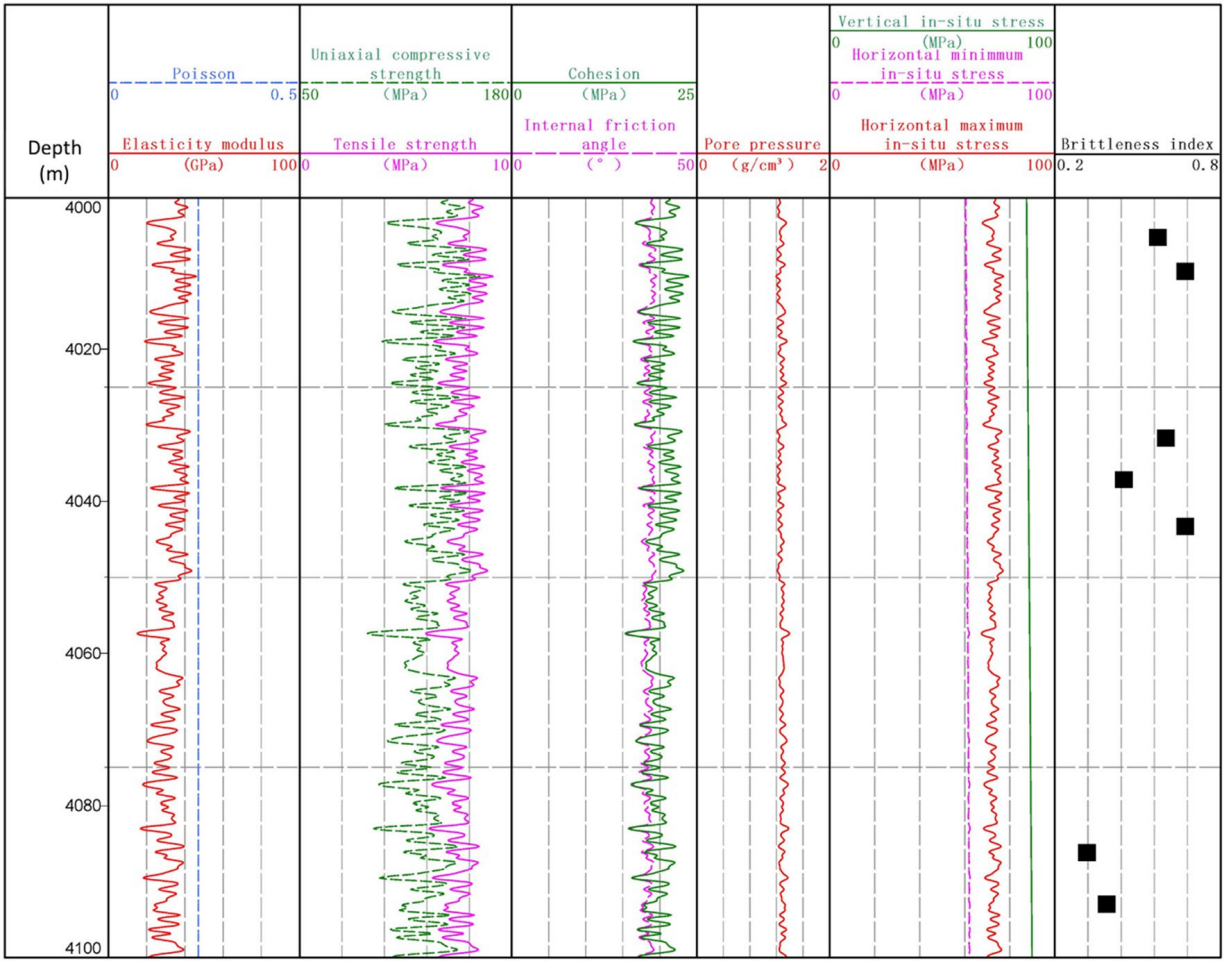
Brittleness index of W4 well.

This new brittleness index has been applied into shale formation of W4 well. Core samples have been taken from W4 well. Thus, brittleness indexes at location of core samples have been calculated using this new index, shown as Fig. 16. It is shown that brittleness index at 4000–4050 m depth is relatively higher that index at 4080–4100 m depth. Average brittleness index is 0.56 at 4000–4050 m depth. In contrast, at 4080–4100 m depth, brittleness index is merely 0.33. According to hydraulic fracturing fracture from micro-seismic monitoring, fracture volume at 4000–4050 m depth is larger than that at 4080–4100 m depth, proving the practicability of brittleness index.

## Conclusion

1. Shale mechanical characteristics have been fully analyzed. Compressive strength has decline with increasing silicate and calcium mineral since quartz, potash feldspar are comparatively strong strength mineral. On the other hand, clay, as relatively lower strength mineral, is adverse to shale strength. Also, high content of silicate mineral lead to large elastic module and small Poisson ratio. In contrast, clay normally has plastic property. Its increasing content lead to small module and large Poisson ratio. Confined pressure can increase compressive strength because it adds up lateral braced force. With increasing bedding plane angle, shale strength will have decline at approximately 55°.
2. A new shale brittleness index has been established based on fully axial and radial stress–strain curve in elastic, plastic and post-peak stage. Its accuracy has been proved by shale failure degree and its correlation with existing brittleness evaluation method.
3. Based on the changing coefficient of all influence factors, important degree of factors have been analyzed. Elastic module has highest weight coefficients. Mineral content plays a big role in shale brittleness. Weight coefficient of confined pressure is large, indicating external condition is vital for shale brittleness evaluation. Rich bedding plane gives shale strong anisotropy in brittleness. Thus, large weight coefficient of bedding plane angle suggests that bedding plane occurrence has to be considered in shale brittleness evaluation.

## Data Availability

The datasets generated and/or analysed during the current study are not publicly available due to requirement of confidentiality, but are available from the corresponding author on reasonable request.
